# Transvenous Retrograde Thoracic Duct Cannulation in a Porcine Model: A Technical Note on Initial Feasibility

**DOI:** 10.1007/s00270-025-04284-4

**Published:** 2025-12-07

**Authors:** Vanesa Lucas-Cava, Francisco M. Sánchez-Margallo, Fei Sun

**Affiliations:** Centro de Cirugía de Mínima Invasión Jesús Usón, Carretera N-521, km. 41,8, 10071 Cáceres, Spain

**Keywords:** Thoracic duct, Subclavian vein, Transvenous retrograde cannulation, Lymphatic intervention, Preclinical study, Porcine model

## Abstract

**Purpose:**

Thoracic duct embolization (TDE) is a valuable minimally invasive treatment for chylous leaks. Pre-clinical evaluation of emerging embolic materials in appropriate animal models is crucial. This study aimed to establish and assess the technical feasibility of transvenous retrograde thoracic duct cannulation, a key component for TDE, in a porcine model.

**Materials and Methods:**

This prospective feasibility study, with institutional animal care committee approval, used five healthy female Large White pigs (mean weight, 50.2 ± 8.9 kg). Under general anesthesia, interstitial lymphangiography of the left forelimb with Lipiodol was performed for guiding transvenous retrograde cannulation. Following percutaneous femoral vein access, retrograde thoracic duct cannulation was attempted using an angiographic catheter and microcatheter assembly. Post-mortem necropsy examined the thoracic duct junction anatomy.

**Results:**

Interstitial lymphangiography visualized the thoracic duct terminus in three pigs. Selective venography consistently demonstrated the thoracic duct ampulla converging at the junction of the left internal and external jugular veins. The angiographic catheter successfully engaged the thoracic duct ampulla in all five cases. Subsequent microcatheter cannulation of the thoracic duct was achieved in three of five pigs. Necropsy confirmed the thoracic duct ampulla’s location between and caudal to the junction of the left internal and external jugular veins.

**Conclusion:**

Transvenous retrograde cannulation of the thoracic duct is technically feasible in the porcine model, offering a platform for pre-clinical TDE research. Technical refinements are warranted to improve success rates.

**Graphical Abstract:**

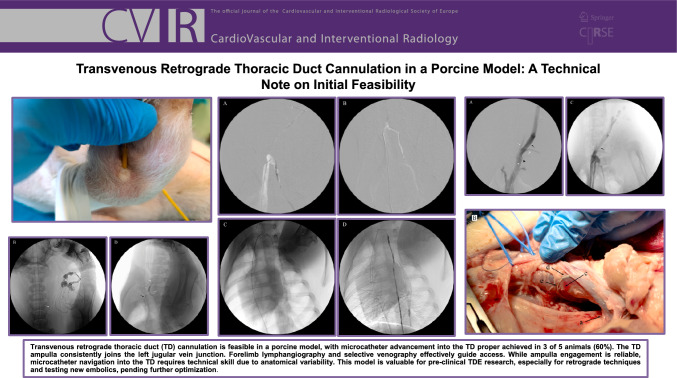

**Supplementary Information:**

The online version contains supplementary material available at 10.1007/s00270-025-04284-4.

## Introduction

Percutaneous thoracic duct embolization (TDE) has become an established minimally invasive and effective alternative to surgery for treating chylous leaks [1, 2]. The conventional TDE approach, pioneered by Cope, involves antegrade transabdominal cannulation of the cisterna chyli (CC) or major retroperitoneal lymphatics, followed by catheterization of the thoracic duct (TD) and embolization [3, 4]. While successful, this antegrade technique faces limitations, including challenges in opacifying or accessing an anatomically variable or small CC, navigating tortuous lymphatics, managing TD occlusions proximal to the leak, and inherent risks of visceral organ penetration [4–7].

To address these challenges, retrograde TD access has been explored clinically. Transvenous retrograde cannulation, typically targeting the TD at its venous confluence, offers an alternative when antegrade access is difficult, contraindicated, or has failed [8–11]. This approach avoids abdominal puncture and can provide direct distal TD access, potentially advantageous for high leaks or proximal obstructions. Direct transcervical retrograde puncture also serves as an alternative technique [5].

Despite the increasing clinical application of retrograde TD interventions in humans, systematic evaluation of these techniques in large animal models remains limited. Such pre-clinical studies may be helpful for refining procedural techniques, understanding model-specific anatomy, and providing a foundation for evaluating novel embolic agents or devices. The porcine model is well-suited for translational interventional radiology research due to its anatomical and physiological similarities to humans [3]. To our knowledge, dedicated studies on the feasibility of transvenous retrograde TD cannulation in swine have not been reported. This study, therefore, aimed to describe and assess the technical feasibility of transvenous retrograde TD cannulation in a porcine model, as a foundational step for developing a reliable pre-clinical TDE research platform.

## Materials and Methods

### Animal Model and Ethical Approval

This prospective study utilized five healthy female Large White pigs with a mean body weight of 50.2 ± 8.9 (SD) kg. All procedures adhered to Spanish national guidelines for animal care and use (Royal Decree 53/2013), Directive 2010/63/EU, and were approved by the Regional Ethics Committee of the Junta de Extremadura, Mérida, Spain (Approval Number: EXP-20240206).

### Anesthesia and Preparation

Animals were fasted overnight with ad libitum water access. Premedication included intramuscular diazepam (0.1 mg/kg) and ketamine (10 mg/kg). Anesthesia was induced with intravenous propofol (2 mg/kg), followed by endotracheal intubation and maintenance with isoflurane (1–3%; IsoFlo, Zoetis, Madrid, Spain) in oxygen. Animals were positioned supine, and the left forelimb and groin areas were aseptically prepared.

### Lipiodol-Based Interstitial Lymphangiography

To guide retrograde cannulation, transpedal interstitial Lipiodol lymphangiography was performed. Under fluoroscopic guidance, 2 mL of Lipiodol [48% w/vol Iodine (480 mg/mL)] (Guerbet, Villepinte, France) was manually injected intradermally at approximately 1 mL/min into each of the three interdigital web spaces of the left forelimb using a 25-gauge hypodermic needle (0.5 × 16 mm; Nipro Medical Europe, Zaventem, Belgium). Intermittent fluoroscopy monitored contrast progression and aimed to prevent venous intravasation. Spot radiographs were obtained post-injection to visualize Lipiodol distribution in efferent lymphatics and the TD outlet.

### Selective Venography and Retrograde Thoracic Duct Cannulation

Percutaneous access to the right femoral vein was obtained using a 7 Fr introducer sheath (Radifocus®, Terumo). A 5 Fr angiographic catheter (Bern, 0.038" × 90 cm, Boston Scientific) and a 0.035" guidewire (Roadrunner, Cook Medical) were advanced into the left brachiocephalic vein. Referencing the Lipiodol opacification of the TD outlet, selective retrograde venography was performed to delineate the anatomy of the left internal jugular, external jugular, and subclavian veins, as well as the TD terminus.

With the animal positioned to achieve a left anterior oblique view (LAO-30°), the angiographic catheter was manipulated to engage the TD ampulla. Thoracic ductography was then performed to visualize the ampulla, genu, and caudal TD. Under roadmap guidance, a microcatheter (Progreat 2.4 Fr, Terumo) paired with a microguidewire (0.016"– 45° angled GT wire, Terumo) was carefully advanced through the TD genu toward the caudal TD. For tortuous routes, lymphatic valves, or fine side branches, the 0.016" GT wire was exchanged for a straight 0.014" microwire (NeuroScout, Cordis) to facilitate distal TD navigation. Thoracic ductography was repeated via the microcatheter upon successful placement.

### Anatomical Study at Necropsy

Immediately following the procedure, animals were euthanized with a lethal dose of potassium chloride (KCl) solution. Necropsy was performed by interventional radiologists and veterinary staff with the animal supine. The left external and internal jugular veins, left subscapular vein, and left brachiocephalic vein were meticulously dissected. The terminal TD, typically beneath the external jugular vein after lateral retraction, was identified. Anatomical details were photographically documented.

## Results

### Lipiodol-Based Interstitial Lymphangiography for TD Terminus Visualization

**Fig. 1:**
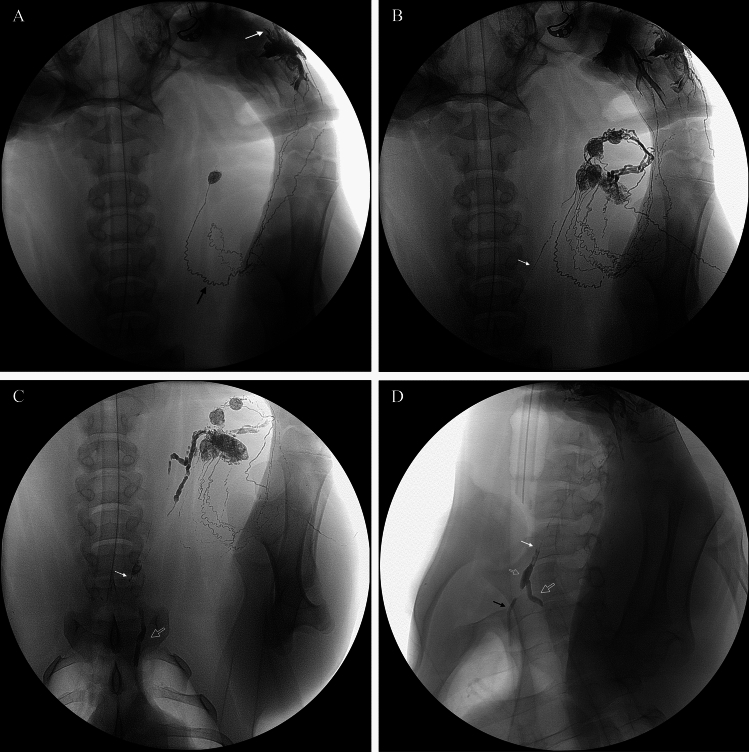
Lipiodol-based interstitial lymphangiography of the left forelimb in pigs, demonstrating sequential opacification. **A** Image at 1 min post-intradermal Lipiodol injection shows initial opacification of lymphatics in the limb and neck, including a dorsal superficial cervical lymph node. The black arrow indicates the afferent lymphatic vessel from the injection site (white arrow) to the node. **B** At 4 min post-injection in the same pig, increased opacification of dorsal superficial cervical lymph nodes is visible, along with their efferent lymphatic vessel (white arrow). **C** At 41 min post-injection, further progression shows opacified dorsal superficial cervical lymph nodes, the efferent lymphatic vessel (white arrow), and the emerging thoracic duct (TD) (open arrow). **D** At 71 min post-injection in another pig, opacification includes the efferent lymphatics (white arrow), the TD ampulla (small open arrow), the thoracic part of TD (large open arrow), and the angiographic catheter (black arrow)

Interstitial lymphangiography of the left forelimb successfully opacified the terminal thoracic duct (TD) in three of the five pigs. The dose of Lipiodol utilized for forelimb interstitial lymphangiography was approximately 0.12 mL/kg (6 mL total per animal; mean body weight, 50.2 kg). In these three animals, the TD ampulla, and its confluence with efferent lymphatics from the left dorsal superficial cervical lymph nodes, became visible fluoroscopically at 41, 71, and 134 min post-Lipiodol injection, respectively (Fig. 1). In the remaining two pigs, lymphangiography failed to delineate the terminal TD; one showed opacification limited to forelimb lymphatics and the dorsal superficial cervical lymph nodes, while the other experienced venous intravasation of contrast.

### Selective Venography and Thoracic Duct Anatomy at the Junction

**Fig. 2:**
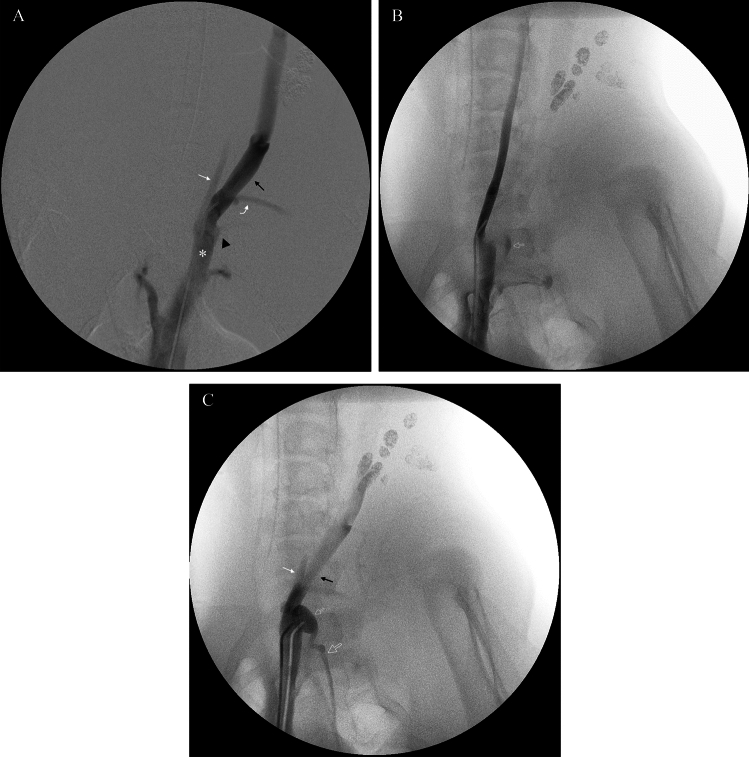
Representative venography images demonstrating local vascular anatomy at the thoracic duct (TD) terminus in pigs. **A** Overview venogram showing major tributaries of the left brachiocephalic vein (asterisk): left internal jugular vein (white arrow), external jugular vein (black arrow), subscapular vein (curved white arrow), and the ostium of the subclavian vein (black arrowhead). **B** Selective venogram of the left internal jugular vein with reflux of contrast medium faintly opacifying a portion of the TD ampulla (small open arrow), as further demonstrated in Supplementary Video 1. **C** Selective venogram more clearly delineating the left internal jugular vein (white arrow), external jugular vein (black arrow), the TD ampulla (small open arrow), and the proximal thoracic part of the TD (large open arrow)

**Fig. 3:**
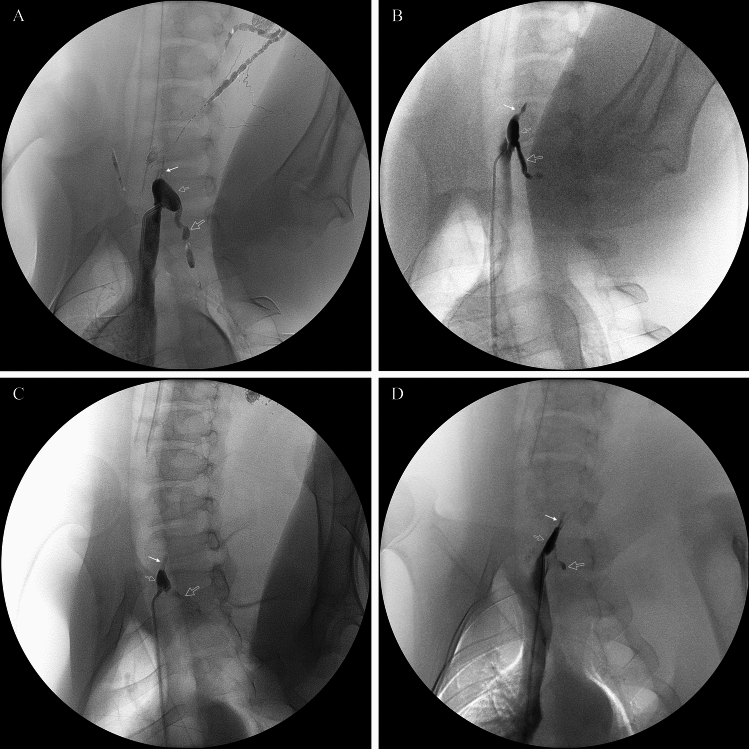
Thoracic ductography images demonstrating morphological variability of the thoracic duct (TD) ampulla in different pigs, with the angiographic catheter tip engaged. Panels (**A**-**D**) illustrate variations in the size and shape of the TD ampulla (small open arrow), the thoracic part of the TD (large open arrow), and the contributing efferent lymphatic vessel from cervical lymph nodes (white arrow). Note that Fig. 3D represents one of the cases where subsequent microcatheter navigation into the thoracic duct proper was not achieved

Selective venography consistently demonstrated that the left brachiocephalic vein was formed by the confluence of the ipsilateral subclavian, external jugular, and internal jugular veins. In all five animals, the TD terminus was identified inserting dorsally at the union of the left external and internal jugular veins (Fig. 2). The TD, ascending from the thoracic cavity, coursed ventrocranially to merge with efferent lymphatics from the left dorsal superficial cervical lymph nodes (between the C7 and T1 vertebral levels). This union formed a distinct TD ampulla which then joined the venous system at the posterior wall of the jugular vein junction. The size and morphology of this TD ampulla exhibited considerable inter-animal variability (Fig. 3), which presented a key technical challenge for subsequent retrograde cannulation, particularly with smaller or atypically shaped ampullae.

### Retrograde Cannulation of the Thoracic Duct: Technical Success

**Fig. 4:**
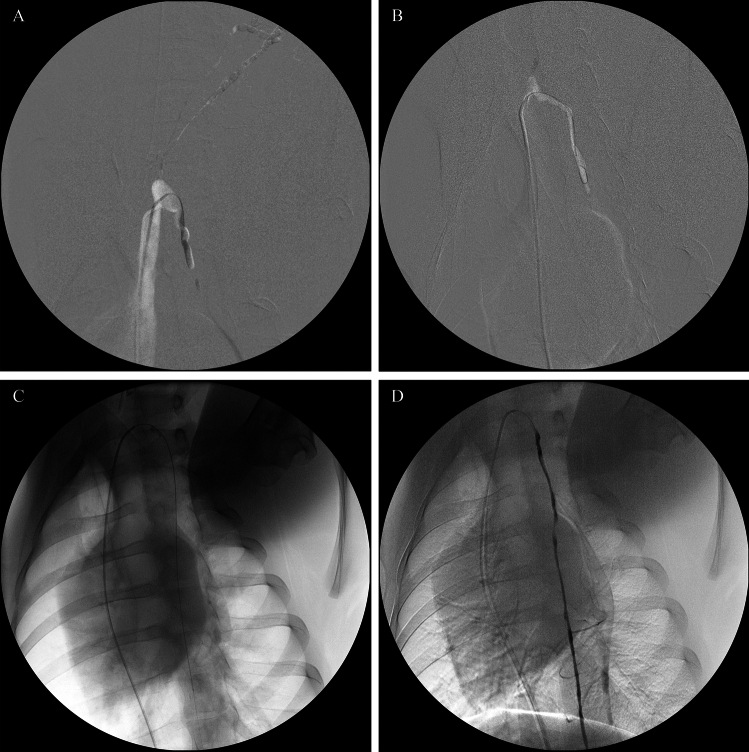
Fluoroscopic images demonstrating procedural manipulations during retrograde cannulation of the thoracic duct (TD) with an X-ray projection of LAO-30°. **A** Under roadmap guidance, a 0.016-inch, 45° angled GT microwire (Terumo) is advanced from a 5 Fr angiographic catheter through the TD genu into the thoracic part of the TD. **B** A 0.014-inch straight microwire (NeuroScout, Cordis) is used to navigate a lymphatic valve within the TD; note the bending of the floppy tip facilitating passage. **C** A microcatheter and microwire assembly is advanced to navigate into the distal TD. **D** Thoracic ductogram performed via the successfully placed microcatheter, opacifying the TD

Technical success was defined as the successful placement of the microcatheter into the thoracic part of the TD, as confirmed by fluoroscopy and/or injection of contrast. A 5 Fr angiographic catheter was successfully engaged into the TD ampulla under fluoroscopic guidance in all five pigs. Subsequent advancement of a microcatheter into the thoracic duct proper was achieved in three of these five animals (Fig. 4). The two instances of unsuccessful microcatheter advancement were attributed to the small size of the ampulla and an acute angulation at the TD’s venous termination. Of the three pigs where interstitial lymphangiography successfully opacified the TD terminus, microcatheter advancement into the thoracic duct proper was achieved in one pig and was unsuccessful in the other two. In the two pigs where lymphangiography failed to delineate the TD terminus, microcatheter advancement into the thoracic duct proper was successful in both cases.

### Anatomical Confirmation at Necropsy

**Fig. 5:**
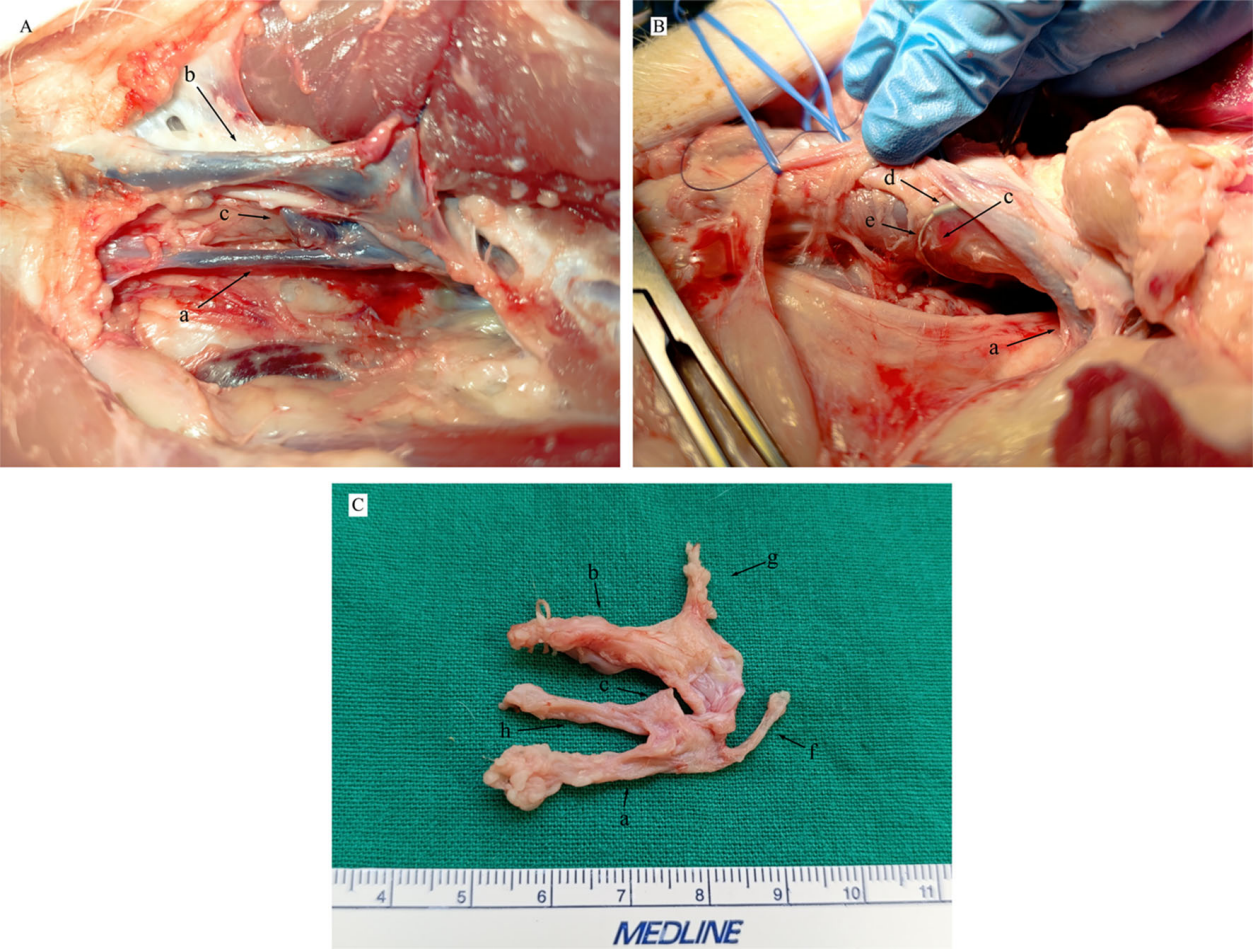
Necropsy photographs demonstrating the local anatomy of the thoracic duct (TD) terminus with the animal in a supine position (cranial to the left). **A** Dissection showing the TD ampulla (c) in its deep position, situated between the left internal jugular vein (a) and the retracted left external jugular vein (b). **B** Further dissection with both the left external jugular vein and internal jugular vein (a) retracted, exposing the TD ampulla (c). An angiographic catheter (d) and guidewire (e), previously placed during the procedure, are visible within the TD. **C** Ventral view of the excised specimen illustrating the relationships: internal jugular vein (a), external jugular vein (b), TD ampulla (c), thoracic part of the TD (f), subscapular vein (g), and an efferent lymphatic vessel (h) joining the TD ampulla

Necropsy findings corroborated the in vivo imaging. The major tributaries to the left brachiocephalic vein at the C7–T1 level were confirmed as the left subclavian, external jugular, internal jugular, and subscapular veins. The TD ampulla, formed by the union of the cranial efferent lymphatic of the left dorsal superficial cervical lymph nodes and the caudal TD trunk, was consistently located beneath the left external jugular vein and dorsolateral to the internal jugular vein, joining the venous system at the junction of these two jugular veins (Fig. 5).

## Discussion

This preliminary study demonstrates the technical feasibility of transvenous retrograde cannulation of the thoracic duct (TD) in a porcine model. While antegrade transabdominal TD access remains a common approach for thoracic duct embolization (TDE) [3, 4], developing alternative access routes is valuable for overcoming its inherent limitations and establishing platforms for pre-clinical research. Our findings suggest that with appropriate imaging guidance and catheter techniques, the porcine TD terminus can be successfully accessed via a transvenous retrograde route.

Accurate anatomical understanding is crucial for technical success. In humans, the TD typically terminates in the internal jugular vein, subclavian vein, or their junction in over 95% of cases, with rarer terminations in other cervical veins [8, 10, 12]. In this limited porcine cohort, the consistent anatomical location of the TD ampulla, inserting dorsally at the junction of the left internal and external jugular veins, was observed. This anatomical consistency, even without prior lymphangiographic TD opacification, appeared to provide a reproducible reference point for experimental approaches. Notably, the plexiform cervical TD anatomy described in humans [10] was not observed in our study. The observed more uniform terminal TD structure in this limited porcine cohort, in contrast to the plexiform cervical TD anatomy described in some human series, suggests potential anatomical differences that warrant further investigation with larger cohorts.

Effective localization of the TD terminus is the initial critical step. Clinical retrograde approaches often employ conventional transpedal or ultrasound-guided intranodal lymphangiography [8, 10], which can be time-consuming or technically demanding. Advanced imaging modalities such as MR thoracic ductography (MRTD) also offer noninvasive visualization of the TD and its venous connections, proving valuable for pre-procedural planning [13].

For the initial localization of the TD terminus, we employed interstitial lymphangiography of the left forelimb, a technique novel for this specific central lymphatic application to our knowledge. This approach successfully opacified the TD outlet in 3 of 5 pigs, showing promise with its relative ease, potentially lower Lipiodol dosage, and quicker opacification of the TD ampulla compared to traditional pedal routes in swine. While the broader utility of interstitial lymphangiography for peripheral lymphatic assessment is gaining attention, its application for visualizing central lymphatic structures varies with technique and animal model. For instance, a recent study utilizing interstitial Lipiodol injection in a swine model successfully imaged peripheral hindlimb lymphatics and superficial inguinal lymph nodes, but reported challenges in visualizing more central structures like the cisterna chyli or the main thoracic duct [14]. This highlights that while peripheral interstitial lymphangiography may hold promise for diagnosing localized postoperative lymphatic leakage, our findings suggest this simpler forelimb interstitial technique, by effectively visualizing the TD terminus, could serve as a valuable alternative or complementary tool for guiding retrograde TD cannulation. However, its direct clinical translatability to human practice warrants further dedicated investigation.

The dose of Lipiodol utilized for forelimb interstitial lymphangiography was approximately 0.12 mL/kg (6 mL total per animal; mean body weight, 50.2 kg). This is lower than typical intranodal lymphangiography doses reported in humans, which often range around 0.25 mL/kg for specific regions. This difference suggests a potential advantage for interstitial approaches if they prove effective at lower doses, offering a less invasive alternative. However, direct dose translation between species is complex due to physiological and anatomical differences, requiring further investigation into optimal dosing strategies for human application. Furthermore, it is crucial to acknowledge that this study was conducted in healthy porcine models with presumed normal thoracic ducts. The lymphatic anatomy and function in diseased states, such as those involving chylous leaks, may differ significantly. Additionally, the potential effects of anesthetic agents, particularly isoflurane, on lymphatic contractility and drainage patterns could influence lymphangiographic opacification and warrant further dedicated research [15].

Selective venography is essential to delineate the TD ampulla amidst the complex confluence of the internal and external jugular veins. We found the LAO-30° fluoroscopic view beneficial for separating these venous structures and elongating the TD ampulla and genu. Advanced imaging, such as 3D rotational venography or cone-beam CT, could further enhance pre-procedural planning and intra-procedural guidance by improving 3D spatial understanding. Engagement of the TD ampulla with a 5 Fr angiographic catheter was achieved in all animals, a crucial step for accessing the thoracic TD, and recently identified as a significant prognostic factor for successful transvenous retrograde cannulation [16].

Despite consistent ampulla engagement, subsequent microcatheter advancement into the TD proper was successful in only 3 of 5 cases. This highlights the technical challenge of negotiating the often-acute angle of the TD genu and navigating past lymphatic valves. Although the technical success rate of transvenous retrograde canalization of the TD in the present study was similar to the findings of about 70% as reported in clinical reports [10, 15], the small number of animals (n = 5) in this study was not sufficient to provide adequate validation. A large cohort animal study remains necessary to further investigate this matter. Ampulla size and morphology variability, observed both radiographically and at necropsy, were significant contributing factors, with smaller ampullae and acute insertion angles posing the primary obstacles. The judicious selection of appropriately shaped microcatheters and steerable low-profile microwires (e.g., angled GT wire, straight NeuroScout) is critical for overcoming these hurdles. Based on our experience, the use of steerable, angled-tip guidewires was beneficial in navigating challenging ampulla anatomies. Once the microcatheter tip has passed over the ampulla and is navigating in the thoracic part of the TD, it is advisable to use a guidewire with a longer soft straight tip rather than a steerable, angled-tip guidewire. This will allow the guidewire to safely navigate through the lymphatic valves in the TD and proceed directly into the main trunk, while avoiding the fine side branches. Further technical optimization is warranted, including the development of more flexible and highly steerable microcatheters, such as the SwiftNINJA system which offers adjustable tip angulation [17], to enhance navigation through the challenging, often acutely angled lymphatic anatomy. Beyond technical refinements in device design, future research could explore pharmacological adjuncts, such as the potential role of intralymphatic vasodilators like calcium channel blockers (CCBs), to relax lymphatic smooth muscle and facilitate catheter passage in cases of challenging ampulla morphology [18].

In summary, the visualization of the TD-JV junction using interstitial lymphangiography serves as a fundamental and valuable first step. Secondly, successful engagement of the TD ampulla with an angiographic catheter is crucial. Finally, the proper selection of microcatheters and microwires, coupled with meticulous manipulation, is essential for successful retrograde thoracic duct cannulation.

This study has several limitations inherent to a preliminary feasibility investigation. First, its small sample size necessitates a larger cohort to robustly define success rates and identify predictors of technical difficulty. Second, the study focused solely on cannulation; actual thoracic duct embolization (TDE) was not performed, which would introduce additional technical considerations. Third, the use of healthy animals may not fully replicate the potentially altered lymphatic anatomy or tissue friability found in diseased states. Finally, a notable limitation is the lack of detailed documentation regarding radiation metrics (e.g., fluoroscopy time and total dose) for the cannulation procedures.

## Conclusion

Transvenous retrograde cannulation of the thoracic duct is technically feasible in the porcine model, establishing a valuable large animal platform for preclinical research in lymphatic interventions. Further technical optimization is warranted to improve the consistency of successful cannulation and broaden the application of this approach.

## Supplementary Information

Below is the link to the electronic supplementary material.

Supplementary file1: Dynamic fluoroscopic sequence demonstrating true reflux of contrast medium from a selective left internal jugular vein venogram into the thoracic duct ampulla during venography.
